# Loss of FAT1 drives cyclophosphamide resistance in breast cancer via the Wnt/β-Catenin pathway

**DOI:** 10.7150/ijbs.117161

**Published:** 2026-01-01

**Authors:** Lijing Zhong, Dongyan Cao, Chen Zheng, Liyi Zhang, Yuxuan Xu, Shasha Zhao, Xiao Liu, Guiying Wei, Gengming Niu, Heming Xu, Xuan Tang, Jingyuan Wen, Paul W R Harris, Jian Zhang, Aina He, Dongxi Xiang

**Affiliations:** 1 State Key Laboratory of Systems Medicine for Cancer, Shanghai Cancer Institute, Department of Biliary-Pancreatic Surgery, Department of Gastrointestinal Surgery, Renji Hospital, Shanghai Jiao Tong University School of Medicine, Shanghai, People's Republic of China.; 2 Department of Breast Surgery, the First Affiliated Hospital of Xiamen University, School of Medicine, Xiamen University, Xiamen, People's Republic of China.; 3 Shanghai OneTar Biomedicine, Shanghai, People's Republic of China.; 4 Department of Oncology, the Sixth People's Hospital Affiliated to Shanghai Jiaotong University School of Medicine, Shanghai, People's Republic of China.; 5 School of Pharmacy, Faculty of Medical and Health Sciences, and Maurice Wilkins Centre for Molecular Biodiversity. The University of Auckland, Auckland, New Zealand.; 6 School of Biological Sciences, School of Chemical Sciences and Maurice Wilkins Centre for Molecular Biodiversity. The University of Auckland, Auckland, New Zealand.; 7 Department of Phase I Clinical Trial Center, Department of Medical Oncology, Fudan University Shanghai Cancer Center, Shanghai, People's Republic of China.; 8 Shanghai Key Laboratory of Cancer System Regulation and Clinical Translation, Jiading Branch, Renji Hospital, Shanghai Jiao Tong University School of Medicine, Shanghai, People's Republic of China.; 9 Key Laboratory of Early Prevention and Treatment for Regional High Frequency Tumor (Guangxi Medical University), Ministry of Education, Nanning, People's Republic of China.; 10 Jiaxing Organoid Center, Jiaxing, Zhejiang, People's Republic of China.

**Keywords:** tumor organoids, breast cancer, *FAT1*, drug screening test, precision medicine

## Abstract

Drug resistance remains a major obstacle to successful chemotherapy, leading to treatment failure and tumor recurrence. Recent studies indicate that mutations in FAT Atypical Cadherin 1 (*FAT1*) contribute to drug resistance in cancer cells. However, the precise role and underlying mechanisms of *FAT1* in breast cancer (BC) remain insufficiently explored. Here, we conducted a comprehensive genomic and transcriptomic analysis, identifying* FAT1* as a crucial tumor suppressor gene in BC. Our study demonstrates that genomic alterations in *FAT1* are associated with the Wnt/β-catenin pathway activation. We further show that *FAT1* loss induces cyclophosphamide (CTX) resistance and leads to the upregulation of the Wnt signaling cascade, accompanied by the accumulation of CTNNB1 transcription factors. Notably, combination therapy effectively alleviates drug resistance by suppressing the Wnt pathway. These findings highlight the critical role of *FAT1* loss in mediating CTX resistance in BC and provide insights into potential therapeutic strategies targeting the Wnt pathway.

## Introduction

Breast cancer (BC) has recently surpassed lung cancer as the most commonly diagnosed malignancy in women worldwide 1. Despite significant advances in early detection and treatment, BC remains a leading cause of cancer-related mortality 2. Chemotherapy remains a cornerstone in BC treatment, significantly reducing recurrence and metastasis risk while improving survival in advanced cases 3. Standard regimens, including anthracyclines and taxanes, are frequently combined with cyclophosphamide (CTX). Moreover, molecular-targeted therapies such as trastuzumab, pertuzumab, CDK4/6 inhibitor (CKI), and PARP inhibitor (PARPi) are employed with chemotherapy 4, 5.

However, primary and acquired drug resistance continues a major obstacle to successful treatment, contributing to disease progression and poor prognosis 6. Among the various genetic alterations implicated in BC, FAT Atypical Cadherin 1 (*FAT1*) mutations have emerged as critical determinants of tumor behavior and therapeutic response 7, 8. *FAT1*, a member of the cadherin superfamily, is known to function as a tumor suppressor, and its loss has been associated with enhanced cell proliferation and metastatic potential 9-11. Recent studies suggest that *FAT1* mutations may lead to aberrant activation of the Wnt signaling pathway, a key regulator of cell growth, differentiation, and therapy resistance 10, 12-14. However, the precise mechanisms by which *FAT1* modulates drug resistance remain poorly defined.

In this study, we investigated *FAT1* genomic alterations using patient-derived organoids (PDOs) and assessed the impact of *FAT1* loss on CTX resistance. We further explored the role of combination therapy in overcoming drug resistance through Wnt pathway inhibition. Our findings offer novel insights into the molecular mechanisms underlying CTX chemoresistance in BC and identify potential therapeutic avenues for patients harboring *FAT1* mutations.

## Results

### BC PDOs retain characteristics of primary tumors

**Figure 1:**
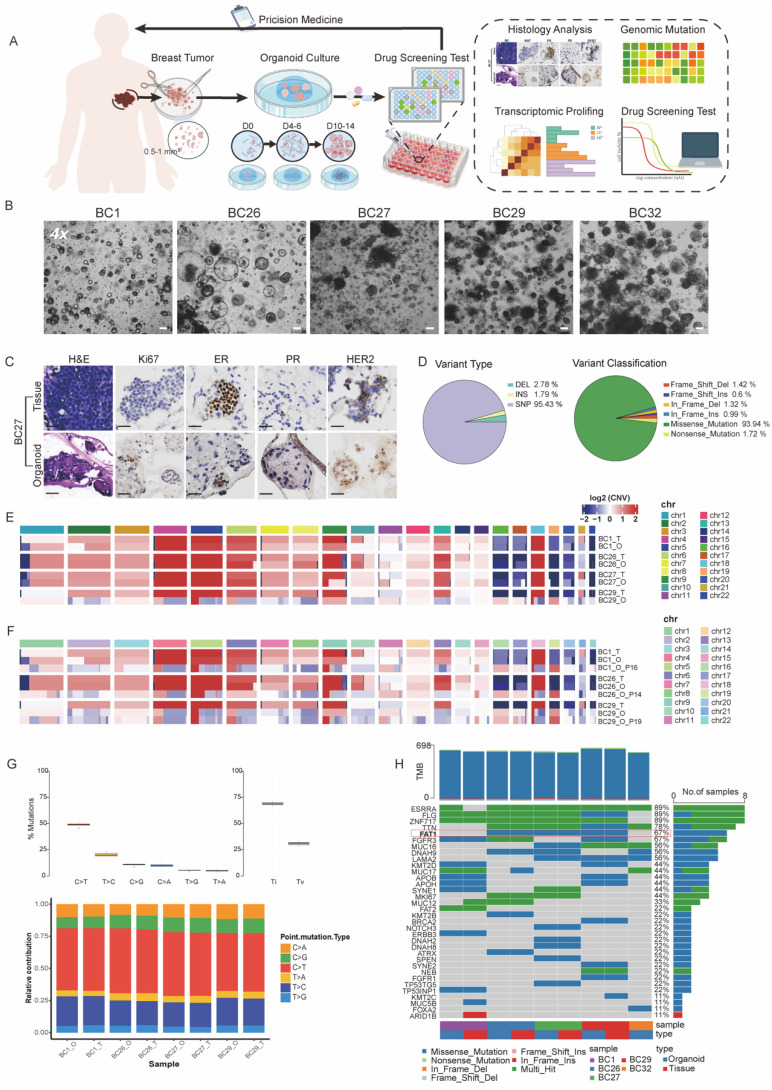
** PDOs recapitulate the histopathologic and genetic characteristics of parental tissue.** (A) Schematic overview of a precision oncology workflow. PDOs are established from breast tumor tissues and typically mature within days 10-14. Once matured, PDOs undergo downstream analyses to guide personalized treatment strategies. (B) Bright-field microscopy images of PDOs established from BC samples of five individual patients (BC1, BC26, BC27, BC29, and BC32). Scale bar: 100 µm. (C) H&E and IHC staining (Ki67, ER, PR, HER2) confirm that BC27 maintain the molecule feature of the original tissue. Scale bar: 100 µm. (D) Summary of mutation types and variant classifications in PDOs. The left pie chart shows the distribution of variant types: single nucleotide polymorphisms (SNP, 95.43%), insertions (INS, 1.79%) and deletions (DEL, 2.78%). The right chart categorizes variants, including frame-shift deletions (1.42%) / insertions (0.6%), in-frame deletions (1.32%) / insertions (0.99%), missense mutations (93.94%) and nonsense mutations (1.72%). (E) Heatmap of CNVs analysis reveals genomic alterations across chromosomes in individual genomes. (F) CNV patterns remain stable after more than 10 passages, as shown by comparative heatmaps. (G) Ti (Transition) / Tv (Transversions) ratios are displayed across samples, and mutation spectra indicate that PDOs closely recapitulate the mutational landscape of the original tumor tissues. Sample labels follow the format: BC1_T for breast cancer tissue and BC1_O for the corresponding organoid. (H) Overview of somatic mutations identified in tissue and paired PDOs. The heatmap displays selected gene mutations across samples, with a focus on those harboring *FAT1* mutations. The adjacent bar graph summarizes the frequency of mutation types.

PDOs were characterized to maintain the histological features, genomic profiles, and tumor heterogeneity of primary tissue 15-18. Accordingly, we successfully established individual PDOs from biopsy samples of BC patients (Figure 1A). BC1 and BC29 showed small-solid morphologies, while BC27 and BC32 displayed denser structures. Notably, BC26 presented a hollow-cystic morphology (Figure 1B). Each PDO exhibited unique formation rate, proliferation index, and growth size. Specifically, BC26 and BC32 demonstrated faster organoid formation, indicating a more efficient growth process (Figure S1A). In contrast, BC27 had the lowest proliferation index, suggesting limited growth potential (Figure S1B). Over the six-day period, BC26 became the largest size among all PDOs, followed in size by BC32, BC29, BC27 and BC1 (Figure S1C). These individual variations reflect the heterogeneity of tumors and the diversity among patients. Haematoxylin and eosin (H&E) and immunohistochemical (IHC) staining confirmed that PDOs maintained key histological features and biomarker expression, including Ki-67, estrogen receptor (ER), progesterone receptor (PR), and human epidermal growth factor receptor 2 (HER2), from the parental tissues (Figure 1C; Figure S1D). PDOs maintained stable histoarchitecture and phenotypic features during long-term culture. Consistently, BC1 retained its dense morphology, while BC26 preserved it's a lumen-like structure (Figure S1E).

To evaluate the extent to which PDOs recapitulate the molecular characteristics of their corresponding primary tumors, Whole Exome Sequencing (WES) was conducted to assess their genomic concordance. Consistent with previous WES studies, the results revealed that the missense mutations (93.94%) were most common, with smaller proportions of nonsense mutations, frameshift insertions/deletions, and in-frame variants. Among variant classifications, single-nucleotide polymorphisms (SNPs) (95.43%) were the predominant variation type across all samples followed by deletions (DEL, 2.78%) and insertions (INS, 1.79%) (Figure 1D) 19, 20. Copy number variations (CNVs) in PDOs closely mirrored the those in the primary tumors, exhibiting similar patterns of chromosomes amplification and deletion (Figure 1E). Even after extended passaging, the PDOs preserved the majority characteristic in early-passage PDOs (Figure 1F). Additionally, the spectrum of point mutations of PDOs and parental tumor were highly consistent, with C>T and T>C substitutions being the most dominant types (Figure 1G). Further analysis of COSMIC mutational signatures revealed that the PDOs retained the majority of the mutational signatures (Figure S1F). We also identified several mutations in oncogenes (e.g., *ESR1*, *FGFR3*, *MKI67*, *NRG1*, *ROS1*) and tumor suppressor genes (e.g., *ALK*, *AXIN2*, *BRCA2*, *FAT1*, *KMT2B*, *KMT2D*, *SPEN*) (Figure 1H). In summary, PDOs effectively preserved the histological and genomic features of their parental tumors, confirming their reliability as BC models.

### Drug screening assay in PDOs

**Figure 2:**
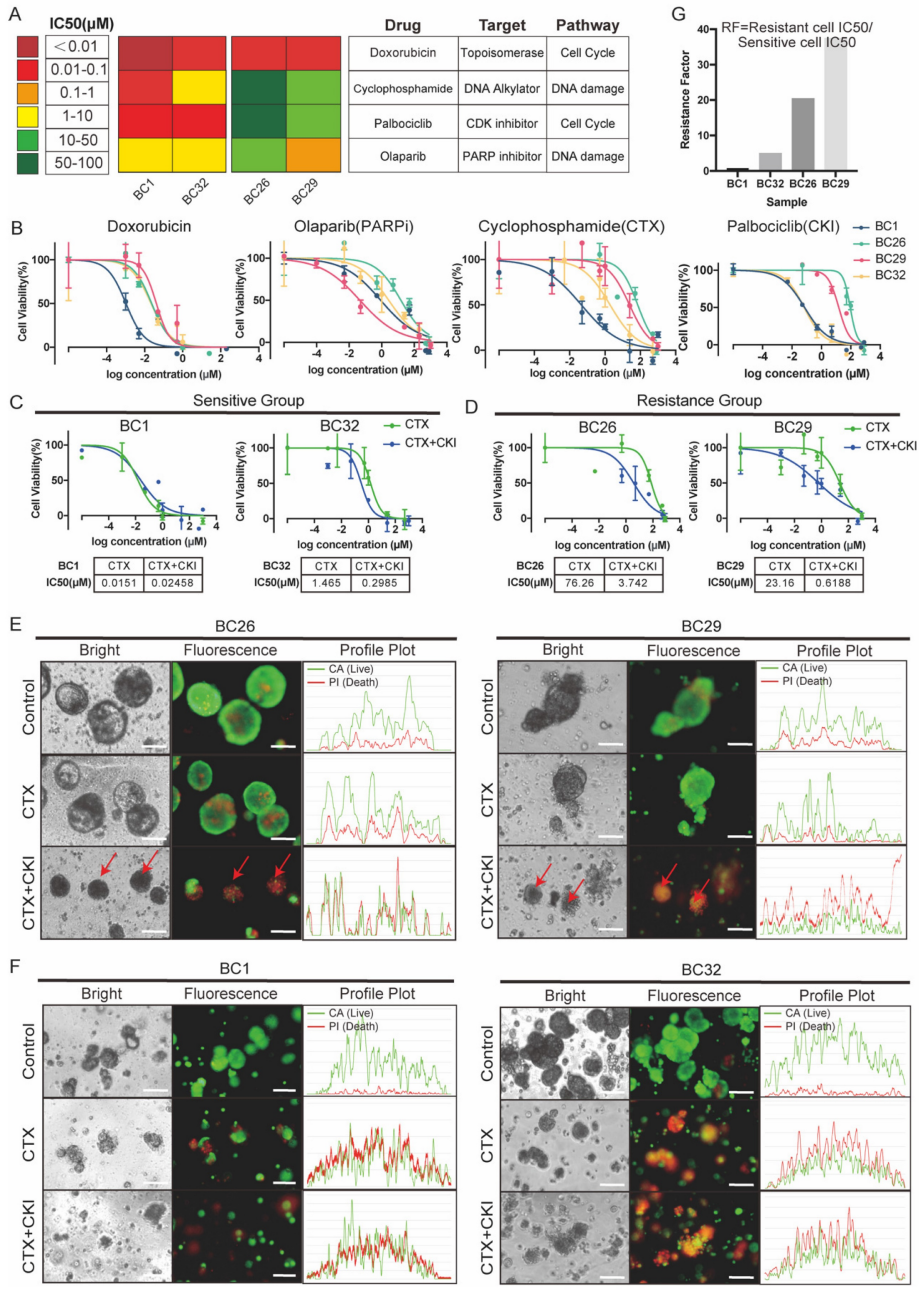
** Drug screening test in PDOs.** (A)The heatmap illustrates IC₅₀ values (μM) of four drugs across PDOs, indicated by color gradients. The corresponding drug targets and associated pathways are listed on the right. (B)Dose-response curves of four different single drugs in PDOs. Error bars: ±s.d, n=2. (C, D) A combination treatment was performed on four PDOs. Each curve represents a different PDO line (BC1, BC26, BC29, BC32). Error bars: ±s.d, n=2. (E, F) Images of PDOs stained with Calcein/PI (Red: dead cell, Green: live cell) under control, CTX and CTX+CKI treatments. Red arrows highlight regions of increased cell death. Scale bar: 100 µm. (G) Bar graph of resistance factor (RF), calculated as the ratio of IC₅₀ values between CKI+CTX combination and CTX monotherapy across PDO samples. Higher RF indicates stronger resistance to CTX alone.

PDOs are increasingly used to model individualized drug responses *in vitro*. Taxanes and anthracyclines, in combination with CTX, commonly known as TAC or AC, are essential first-line chemotherapy regimens in BC treatment 21. To evaluate the utility of PDOs in modeling clinical drug responses, we tested the sensitivity of four PDOs (BC1, BC26, BC29, and BC32) to a panel of chemotherapeutic and targeted agents, including doxorubicin, phosphoramide mustard (active metabolite of CTX), palbociclib (CKI), and olaparib (PARPi) (Figure 2A).

Drug sensitivity was quantitatively assessed using half-maximal inhibitory concentration (IC₅₀) values. Our drug screening results demonstrated PDOs were highly sensitive to doxorubicin at concentrations below 0.1 μM, highlighting its strong therapeutic efficacy in clinical (Figure 2B). Olaparib, a PARPi used in the treatment of BRCA-mutated cancers, demonstrated enhanced toxicity in BC29, which harbored a missense mutation in BRCA2 (Figure 1F, 2B) 22. We also conducted drug screening using CTX and CKI, considering their relevance as commonly used. Specifically, BC26 (CTX, CKI < 100 μM) and BC29 (CTX, CKI < 50 μM) exhibited resistance to single-agent treatment, while BC1 (CTX, CKI < 0.1 μM) and BC32 (CTX < 10 μM, CKI < 0.1 μM) showed relatively higher sensitivity (Figure 2A, 2B). Thus, we categorized PDOs into resistant (BC26 and BC29) and sensitive (BC1 and BC32) groups (Figure 2C).

Combination therapies are essential for combating cancer 23, we treated PDOs with a combination of CTX and CKI (Figure 2C). The results revealed a distinct response pattern between the two groups, with the sensitive group consistently responding well to both monotherapy and combination treatment. In contrast, the resistant group displayed limited response to monotherapy but responded significantly better to combination therapy, with IC₅₀ values reduced by approximately 20- to 40-fold (Figure 2C, D, and G). Live/dead cell staining further confirmed these findings, showing a notable increase in dead cells in the resistant group after combination therapy (Figure 2E). In contrast, the sensitive group exhibited a predominance of dead cells under both treatment conditions, indicating inherent sensitivity to the therapies (Figure 2F). Collectively, these results suggest that combination therapy is more effective than single-agent treatments in the resistant group.

### Transcriptomic analysis of mono- and combination therapy in PDOs

**Figure 3:**
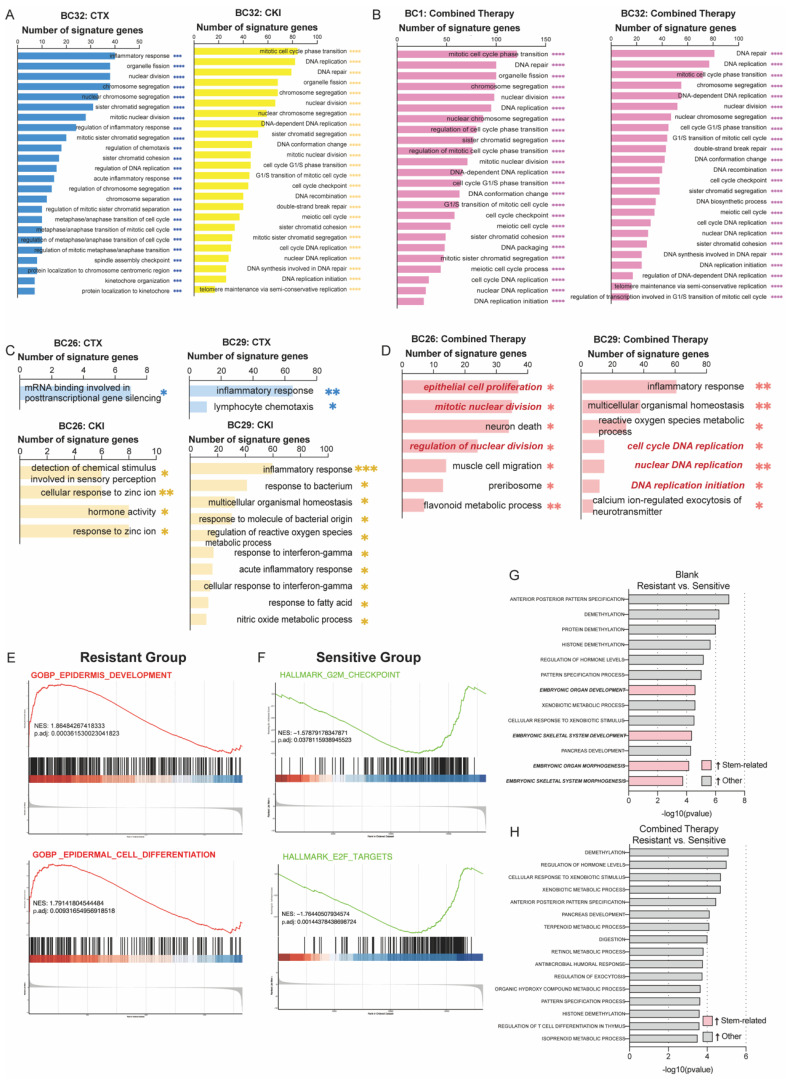
**Bulk-RNA analysis reveals different responses to monotherapy and combined therapy across the sensitive (BC1, BC32) and the resistant group (BC26, BC29).** (A-B) GO enrichment analysis reveals both monotherapy (CTX or CKI) (e.g. BC32) and combined therapy (CTX+CKI) induce strong responses in the sensitive group. (C-D) In the resistant group, monotherapy (CTX or CKI) induces minimal pathway activation, while combination therapy (CTX+CKI) leads to a marked increase, especially in DNA replication and cell cycle progression (highlighted in red). (E-F) GSEA plots illustrate baseline transcriptional differences prior to treatment. In the resistant group, epithelial cell proliferation-related pathways are activated, while cell-cycle related processes dominate in the sensitive group. (G-H) Comparative GO analysis under untreated (G) and combination therapy (H) conditions. Stem-related pathways (pink) are enriched in the resistant group but disappear after combination therapy.

To explore the mechanisms underlying the efficacy of combined therapy in overcoming drug resistance, we performed bulk RNA sequencing on both treated and untreated PDOs. Gene ontology (GO) enrichment analysis showed that pathways related to DNA replication and cell cycle regulation in the sensitive group were significantly downregulated following treatment with either CTX or CKI (Figure 3A). Combination therapy in the sensitive group further reinforced these effects, with consistent suppression of cell cycle-related pathways such as nuclear DNA replication and muscle cell migration, indicating a sustained therapeutic impact (Figure 3B). In contrast, the resistant group exhibited limited changes under monotherapy, with only a few pathways being affected, suggesting reduced responsiveness (Figures 3C). However, combination therapy led to a marked downregulation of proliferation and DNA replication pathways in the resistant group (Figure 3D).

We also conducted gene set enrichment analysis (GSEA) to compare the untreated resistant and sensitive groups. The hallmark analysis revealed that proliferation-related processes, including epidermis development and cell differentiation, were significantly enriched in the resistant group, suggesting that these cells rely on robust growth mechanisms to survive treatment (Figure 3E). In contrast, the sensitive group showed enrichment in cell cycle regulation pathways, such as the G2M checkpoint and E2F targets, indicating that sensitivity to CTX and CKI may be linked to disrupted cell cycle regulation (Figure 3F). Notably, embryonic-related pathways, such as organ and skeletal system development, were significantly enriched in the resistant group and markedly diminished following combination therapy. This shift indicates a reduction in stem-like cancer cells and an enhancement in drug sensitivity (Figure 3G, 3H). These results emphasize the complementary effects of CTX and CKI, highlighting the therapeutic potential of this combination strategy in cancer treatment.

### *FAT1* mutations and expression levels in BC

Genomic instability in cancer cells leads to genetic alterations that contribute to drug resistance and promote tumorigenesis. Among these, SNPs and CNVs in genes are recognized as major drivers of these processes 24. Among the four PDO samples for drug screening, missense mutations of *FAT1* in the resistant group (BC26 and BC29) were detected based on our previous WES data, but not in the sensitive group (BC1 and BC32).

**Figure 4:**
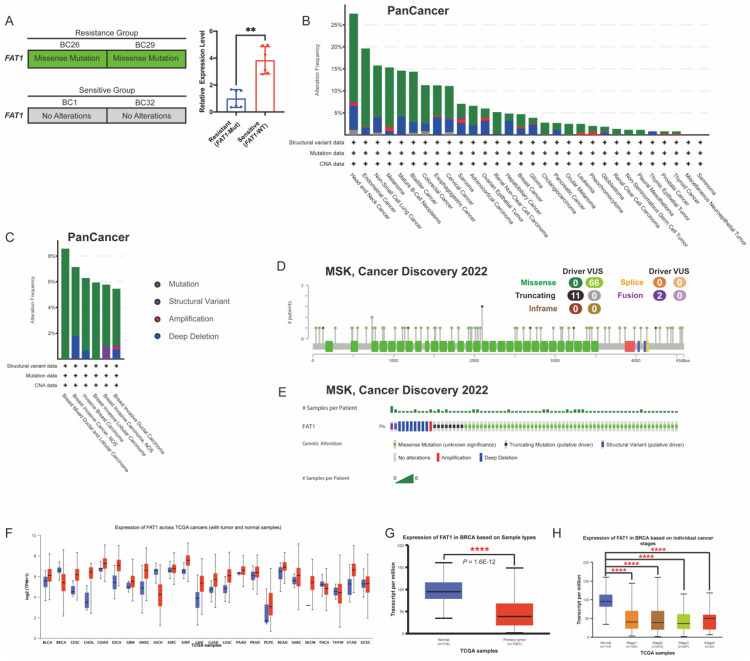
***FAT1* expression levels and mutation landscape in BC.** (A) Missense mutations were detected in the resistant group (BC26, BC29), which also exhibited significantly lower* FAT1* expression compared to the sensitive group (BC1, BC32). n=3, Error Bars: ±s.d. (B) Mutation frequency and types of *FAT1* across tumor types (TCGA, PanCancer Atlas). (C) Distribution of *FAT1* mutation types in BC (TCGA, PanCancer Atlas). (D) Lollipop plot illustrates the location and types in *FAT1* mutation in BC (MSK, Cancer Discovery), with missense mutations being the most prevalent. (E) OncoPrint visualization of *FAT1* (MSK, Cancer Discovery). (F) FAT1 protein level in different tumors (TCGA database). (G-H) Compared to normal breast tissue, FAT1 levels are significantly reduced in BC and are significantly decreased across different stages.

Guided by literature identifying *FAT1* as a frequently mutated gene associated with cancer progression and drug resistance, we focused on *FAT1* (Figure 1H) 7, 14, 25. To further confirm these findings, we quantified *FAT1* mRNA expression using quantitative real-time PCR (qRT-PCR), which revealed significantly lower expression in the resistant group compared to the sensitive ones (Figure 4A).

*FAT1* is frequently mutated across various cancers, and its downregulation has been linked to increased stemness and cisplatin resistance in esophageal squamous cell carcinoma 14. Functional loss of *FAT1* is associated with resistance to CKI through the Hippo signaling pathway, suggesting that *FAT1* plays a key role in modulating drug resistance 26. Given its relevance, we then examined *FAT1* mutations in pan-cancer datasets using cBioPortal, finding that *FAT1* mutations occur in over 10% of nine tumor types (TCGA, PanCancer Atlas) (Figure 4B). The most common *FAT1* mutations were missense (green) and truncating (black), primarily affecting the extracellular and cytoplasmic regions (Figure 4A, 4C-D). In BC, the mutation rate was approximately 7% in a cohort of 1,365 samples (MSK, Cancer Discovery 2022) (Figure 4E), constituting a certain proportion among BC patients.

To further assess expression patterns, we analyzed FAT1 protein levels across 24 tumor types using the UALCAN database. The results confirmed significantly lower FAT1 levels in BC tissues compared to normal breast tissue 11 (Figure 4F-H). These findings suggest that reduced FAT1 expression may contribute to drug resistance and poorer survival outcomes. Accordingly, we classified the sensitive group as *FAT1* wild-type (*FAT1*-WT) and the resistant group as *FAT1* mutant (*FAT1*-Mut) for subsequent analysis.

### *FAT1* loss is associated with CTX resistance

**Figure 5:**
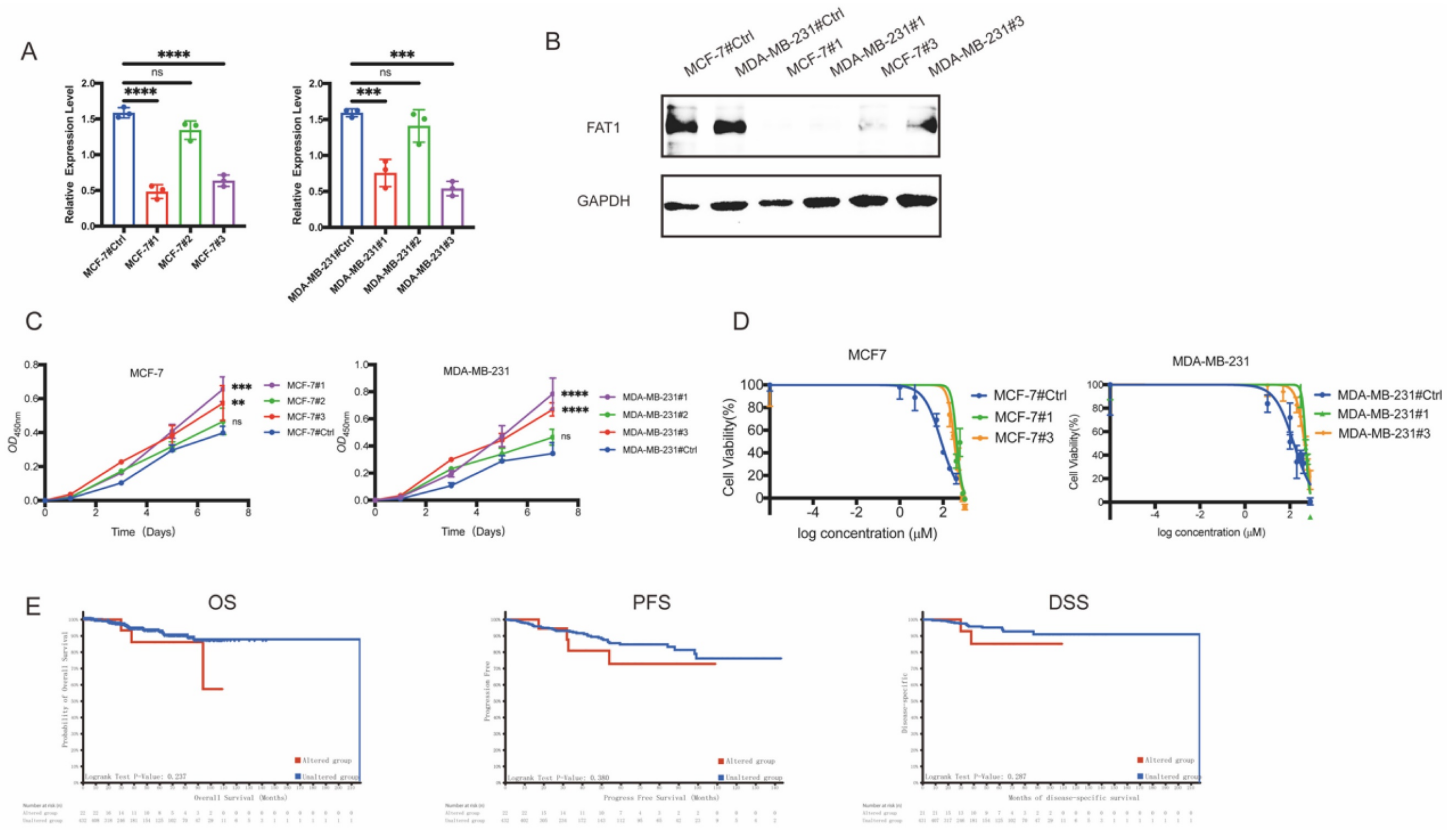
** Low levels of FAT1 contribute to CTX resistance.** (A) Transcriptome level of FAT1 following lentiviral transfection in cell lines (MDA-MB-231, MCF-7). Both group #1 and #3 show significant knockdown efficiency. n=3, Error Bars: ±s.d. (B) Western blot analysis confirms FAT1 protein expression. (C) Cell proliferation assays show enhanced growth in FAT1-KD cells compared to control. n=5, Error Bars: ±s.d. (D) CTX drug sensitivity curves. FAT1-KD cells exhibit significantly higher IC₅₀ values, indicating reduced sensitivity to CTX. n=3, Error Bars: ±s.d. (E) CTX-treated BC patients with *FAT1* mutations show poorer OS (*P* = 0.237), PFS (*P* = 0.380) and DSS (*P* = 0.287) compared to those without mutations (Log-rank test).

To investigate the role of *FAT1* in BC, we engineered MDA-MB-231 and MCF-7 cell lines using siRNAs. In the established FAT1-knockdown (FAT1-KD) cell lines, groups #1 and #3 effectively downregulated FAT1 expression in both MDA-MB-231 and MCF-7 cells, as shown by qRT-PCR (Figure 5A). Western blotting analysis further confirmed decreased FAT1 protein levels in these groups (Figure 5B). Functionally, FAT1-KD significantly accelerated cell proliferation, supporting its role as a tumor suppressor (Figure 5C). We then evaluated the relationship between FAT1-KD and drug resistance. Surprisingly, FAT1-KD cells exhibited increased resistance to CTX. In MCF-7 cells, groups #1 (423.4 μM) and #3 (326.6 μM) showed significantly higher cell viability compared to the control (82.05 μM) under CTX treatment. Similarly, in MDA-MB-231 cells, groups #1 (499.9 μM) and #3 (449.8 μM) exhibited resistance compared to the control (147.3 μM) (Figure 5D). These results indicate that FAT1-KD is associated with reduced sensitivity to CTX.

To further validate our hypothesis, we also analyzed clinical outcomes in a cohort of 454 BC patients treated with CTX from 1,084 samples (TCGA, PanCancer Atlas). Survival analysis revealed that patients with *FAT1* mutations had significantly poorer overall survival (OS), progression-free survival (PFS), and disease-specific survival (DSS) compared to those with wild-type *FAT1* (Figure 5E). These findings suggest that *FAT1* loss contributes to CTX resistance and worse prognosis in BC patients.

### *FAT1* mutation upregulates the Wnt pathway

**Figure 6:**
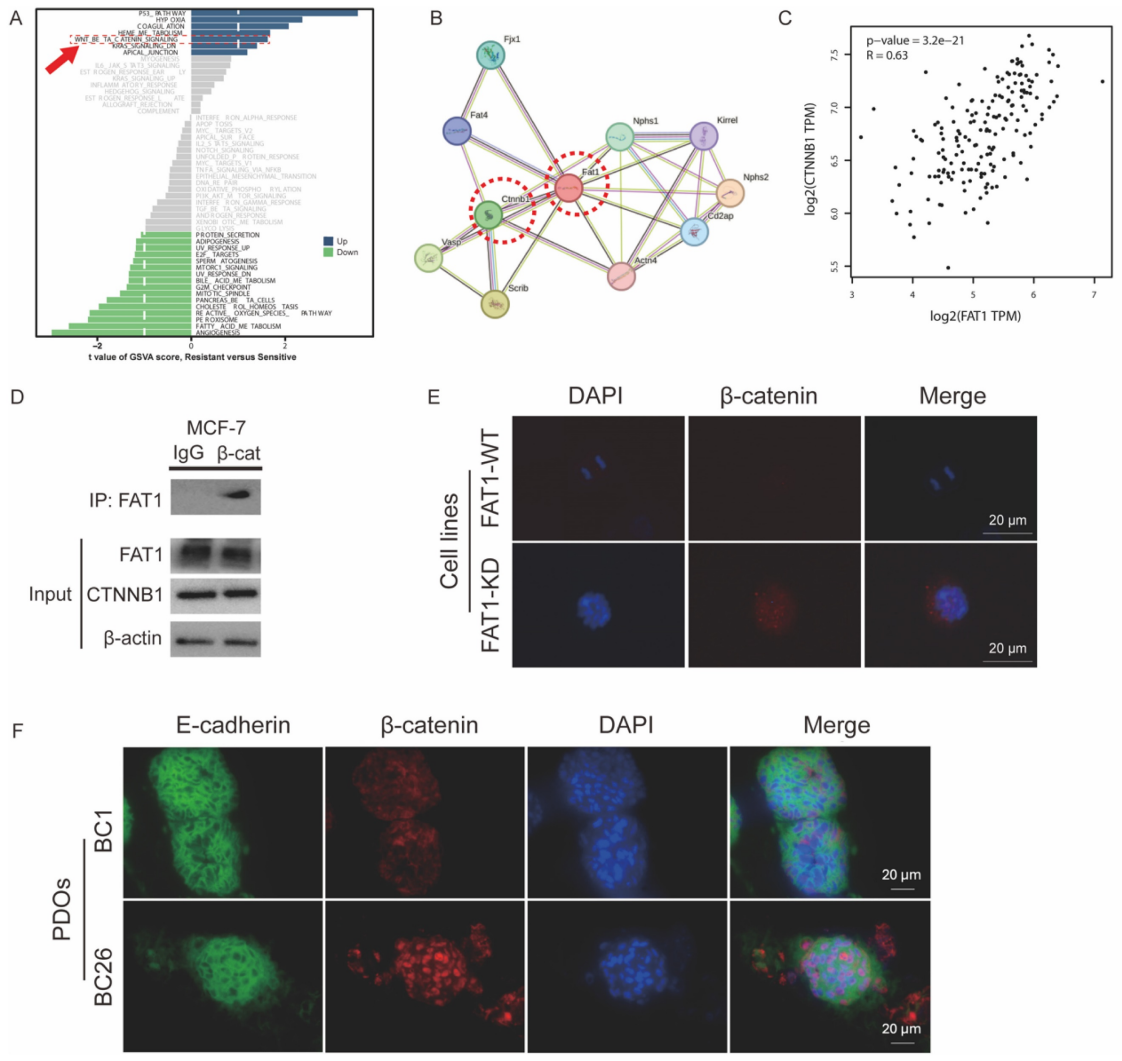
** Mechanism by which FAT1 loss contributes to chemotherapy resistance.** (A) GSVA shows a relative upregulation in the *FAT1*-Mut group (resistant) of the Wnt signaling pathway (red arrow) in GSVA analysis compared to the *FAT1*-WT (sensitive) group. (B) PPI network illustrating the interaction between CTNNB1 and FAT1. (C) Pearson correlation analysis. R=0.63, *P*<0.0001. (D) Co-IP confirms the relationship between FAT1 and β-catenin in MCF-7. IgG was used as a negative control. (E) Immunofluorescence images show increased nuclear localization of β-catenin in *FAT1*-KO of MCF-7. Scale bar: 20 µm. (F) Immunofluorescence images show increased nuclear localization of β-catenin in *FAT1*-Mut BC-PDOs (*vs*. *FAT1*-WT BC-PDOs). Scale bar: 20 µm.

To investigate the mechanisms by which *FAT1* regulates CTX resistance, we compared *FAT1*-Mut and *FAT1*-WT groups. Gene set variation analysis (GSVA) revealed upregulation of the Wnt signaling pathway in the *FAT1*-Mut group even before drug treatment (Figure 6A). The Wnt/β-catenin pathway, a critical regulator of development, tumor proliferation, and drug resistance, has been previously linked to *FAT1* mutations 13, 14.

β-catenin, encoded by the* CTNNB1* gene, is a central component of the canonical Wnt signaling pathway 27. Protein-protein interaction (PPI) analysis indicated a significant interaction between FAT1 and β-catenin, suggesting that FAT1 may modulate Wnt/β-catenin signaling through physical association with β-catenin (Figure 6B).

Pearson correlation analysis showed a moderately strong positive correlation between *FAT1* and *CTNNB1* expression (R = 0.63, P < 0.001) (Figure 6C). Immunofluorescence and co-immunoprecipitation (Co-IP) assays further confirmed the interaction FAT1 and β-catenin in MCF-7 cells (Figure 6D). FAT1-KO cells (Figure 6E) or *FAT1*-Mut PDOs (Figure 6F) enhanced β-catenin nuclear translocation. These results support the hypothesis that loss of FAT1 activates the Wnt/β-catenin pathway in BC, potentially contributing to chemoresistance.

### Combination therapy overcomes drug resistance in *FAT1*-mutant patients

**Figure 7:**
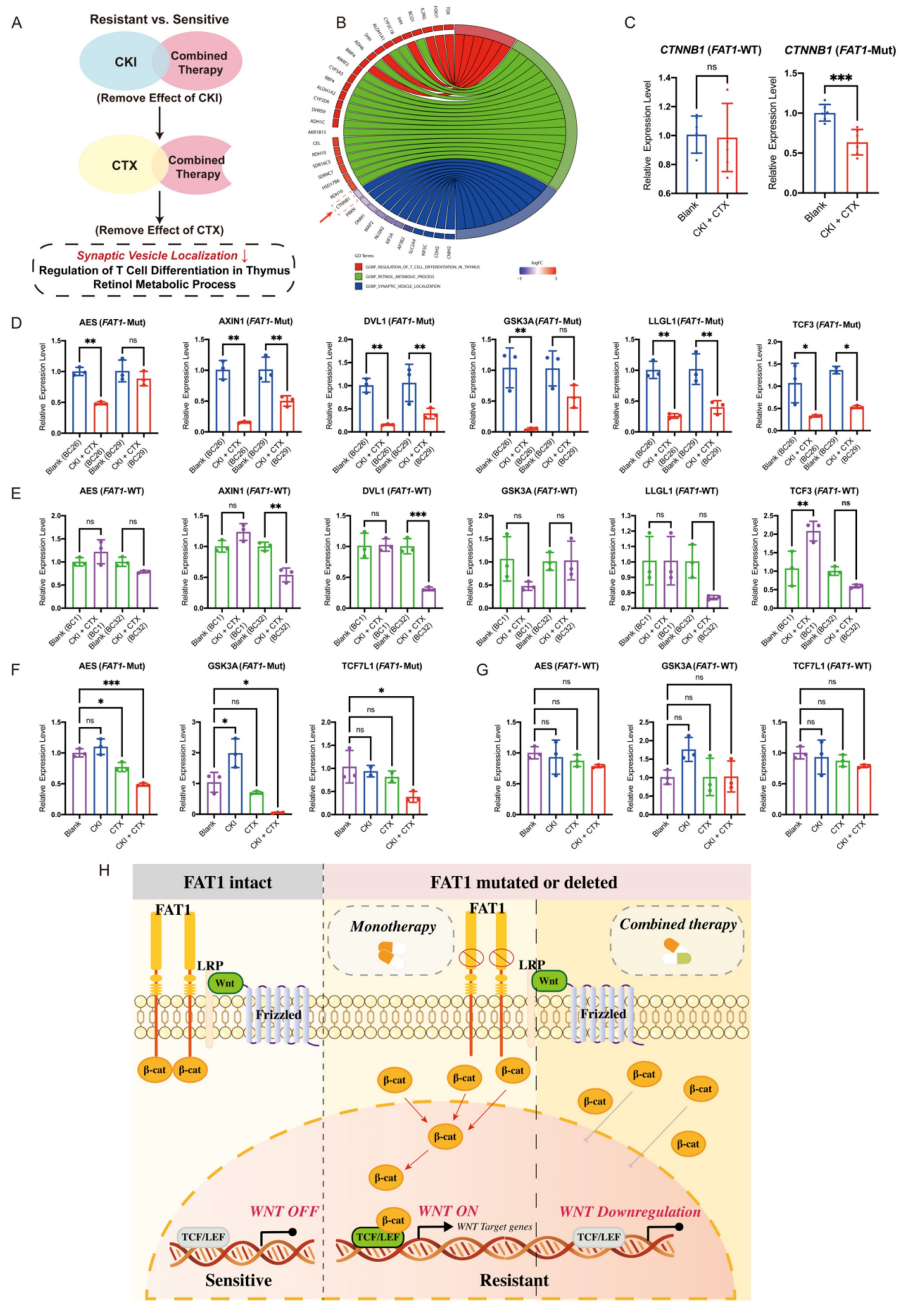
***FAT1* regulates the WNT pathway and promotes CTX resistance.** (A) Schematic overview of pathway enrichment screening. (B) Pathways and gene alterations. *CTNNB1* (red box) was downregulated after combination therapy. (C) *CTNNB1* was downregulated in the *FAT1*-Mut group. n=3, Error bars: ±s.d. (D-E) Expression levels of WNT-related genes in *FAT1*-Mut and *FAT1*-WT group before/ after combination therapy, n=3, Error bars: ±s.d. (F-G) Expression of Wnt-related genes in the *FAT1*-Mut (F) or *FAT1*-WT (G) group before and after using different drugs, n=3, Error bars: ±s.d. (H) Proposed model illustrating the regulatory role of *FAT1*. In *FAT1*-WT cells (left), Wnt signaling is tightly regulated, β-catenin is restricted from nuclear translocation. In *FAT1*-Mut cells (middle), loss of FAT1 leads to β-catenin stabilization and nuclear accumulation, activating Wnt target genes and promoting drug resistance. Combination therapy (right) partially suppresses Wnt signaling and reduces β-catenin activity, thereby alleviating resistance.

To further explore how combination therapy overcomes drug resistance, we analyzed biological pathways enriched in the *FAT1*-Mut group after treatment. GO analysis revealed distinct pathways activated by combination therapy, including synaptic vesicle localization, T cell differentiation regulation in the thymus, and retinol metabolic processes (Figure 7A). Notably, synaptic vesicle localization was the only downregulated pathway, and *CTNNB1* expression within this pathway was significantly reduced (Figure 7B).

Transcriptional analysis confirmed a decrease in *CTNNB1* expression specifically in the *FAT1*-Mut group after combination therapy, with no significant change in the *FAT1*-WT group (Figure 7C). Furthermore, combination therapy downregulated Wnt-related genes (*AES*, *AXIN1*, *DVL1*, *GSK3A*, *LLGL1*, *TCF3*) in the *FAT1*-Mut group (Figure 7D), while these genes remained largely unaffected in the *FAT1*-WT group (Figure 7E). Importantly, monotherapy did not significantly alter Wnt signaling, whereas combination therapy effectively suppressed Wnt-related gene expression in the *FAT1*-Mut group (Figure 7F, 7G).

To sum up, these results suggest that combination therapy alleviates drug resistance in *FAT1*-Mut BC by downregulating the Wnt/β-catenin pathway. In cells with intact *FAT1*, Wnt signaling is tightly regulated, preventing β-catenin accumulation and maintaining treatment sensitivity. In contrast, loss of FAT1 disrupt this regulation results in β-catenin stabilization and nuclear translocation, which activates Wnt target genes and contributes to drug resistance. Combination therapy effectively reduces this effect by suppressing Wnt signaling, thereby restoring drug sensitivity (Figure 7H).

## Discussion

Precision medicine tailor treatments to individual patients, moving away from the traditional one-size-fits-all approach 28. Organoids as 3D self-organized models, closely mimic the physiological functions and structures of original organs, enhancing drug efficacy and safety assessments in drug screening tests (DST) 29-31. PDO-based DST is critical for avoiding ineffective therapies, minimizing side effects, and optimizing time and resource usage 32-38. Several studies have reported predictive accuracies of PDO-based DST,with reported rates exceeding 80% 39. Notably, in the phase III clinical trial (CinClare) involving colorectal cancer patients, PDO-based DST achieved a predictive accuracy of 84.43%, with sensitivity and specificity of 78.01% and 91.97%, respectively 40. These results the clinical value of PDOs in guiding clinical decisions, reducing adverse drug reactions, and alleviating patient suffering, positioning them as a practical and powerful bridge between research and clinical care in precision medicine 41-44.

In this study, we successfully established five PDOs (BC1, BC26, BC27, BC29 and B32) from patients and performed DST with clinically approved first-line chemotherapy drugs, including doxorubicin, CTX, palbociclib, and olaparib. Due to the slow growth of BC27 and the inability to obtain a sufficient number of cells, four other PDOs (BC1, BC26, BC29 and BC32) were used for drug sensitivity testing. Doxorubicin is a cytotoxic anthracycline antibiotic widely used in BC treatment, leading to DNA damage and apoptosis. CTX belongs the class of alkylating agents, commonly used in combination with doxorubicin in AC-based regimens. As CTX is a prodrug that requires hepatic metabolism to generate its active molecule, PM was used in our DST to more accurately reflect its therapeutic activity. Our results exhibited sensitivity to doxorubicin in PDOs, supporting it as a viable first-line chemotherapeutic agent in BC treatment. Olaparib (PARPi) exploits synthetic lethality in BRCA-mutated tumors by blocking DNA repair pathways, leading to accumulation of DNA damage and cancer cell death 5, 45. DST results showed that BC29, which harbors a BRCA mutation, exhibited heightened sensitivity to olaparib, confirming the drug's expected efficacy and the reliability of PDO model. Palbociclib (CKI) is a selective inhibitor of cyclin-dependent kinases CDK4 and CDK6, which blocks cell cycle progression at the G1/S transition and effectively suppresses cancer cell proliferation 46. We also evaluate therapeutic potential of CKI in BC. Based on our experimental data, BC1 and BC32 (sensitive group) were relatively sensitive to both CTX and CKI, whereas BC26 and BC29 (resistant group) exhibited resistance to monotherapy. Prior studies indicate that combination therapy enhances anticancer efficacy by concurrently targeting multiple oncogenic pathways to produce synergistic or additive effects 47. Building on these observations, we hypothesized that combination therapy could yield superior therapeutic outcomes. Notably, our experimental results and bioinformatic analyses supported this hypothesis: the resistant group exhibited improved sensitivity following combination treatment. To further investigate the underlying mechanisms of drug resistance, we integrated genomic and transcriptomic data with literature evidence and focused our analysis on FAT1. As mentioned above, loss of FAT1 function has been shown to promote cancer progression by inducing epithelial-mesenchymal transition (EMT), enhancing stemness, and increasing metastatic potential 9.

CTNNB1 (β-catenin), a key effector in the Wnt/β-catenin pathway, regulates proliferation and differentiation and is involved in tumor progression and drug resistance 48, 49. Specifically, Wnt ligand binding inhibits CTNNB1 degradation, leading to its accumulation and nuclear translocation, where it regulates genes involved in cell survival and proliferation. Previous studies have shown that FAT1 interacts with β-catenin, and FAT1 loss leads to aberrant activation of the Wnt/β-catenin signaling 12, 13. FAT1 inhibits cancer cell growth by binding β-catenin and preventing its nuclear localization, and its loss results in β-catenin stabilization and nuclear translocation, promoting oncogenic Wnt/β-catenin signaling 10,11,36. Our experimental results further demonstrate that functional loss due to FAT1 mutation contributes to CTX resistance in BC patients. Notably, combination therapy with CKI and CTX effectively overcomes this resistance, offering a promising therapeutic strategy. In conclusion, our findings establish a link between FAT1 loss and drug resistance in BC. *FAT1* suppression activates the Wnt/β-catenin pathway, positioning *FAT1* as a potential tumor suppressor and offering a novel therapeutic target for BC treatment.

Moreover, immunotherapy has made significant strides in BC treatment, particularly in triple-negative breast cancer (TNBC), with promising agents such as immune checkpoint inhibitors (ICIs), antibody-drug conjugates (ADCs), and CAR T-cell therapies 50. However, persistent challenges limit widespread application, particularly immune evasion 51. Chemotherapy has been shown to induce immunogenic cell death, activate adaptive immune responses, and enhance antigen presentation, thereby contributing to tumor microenvironment (TME) remodeling. Beyond its cytotoxic effects, chemotherapy may also reduce TME-mediated resistance to tumor-infiltrating lymphocytes, potentially improving the efficacy of ICIs 52. For example, the combination of PD-1 inhibitors such as pembrolizumab with platinum-based chemotherapy has become a first-line treatment option in advanced non-small cell lung cancer (NSCLC), significantly improving OS and PFS 53. In treatment-resistant cases of gastric and pancreatic cancer, the novel antibody PRL3-zumab combined with chemotherapy has shown potential in delaying disease progression 54. In TNBC, the combination of a PD-L1 inhibitor with nab-paclitaxel has demonstrated superior efficacy compared to monotherapy 55. Accordingly, chemo-immunotherapy combinations are increasingly regarded as an effective treatment strategy across multiple cancer types. Meanwhile, understanding the mechanisms behind drug combinations and the role of biomarkers is essential in the development of effective cancer combination therapies. Our work not only indicate that the repurposing of conventional agents in combination with immunotherapy may elicit unanticipated synergistic effects, but also elucidates the underlying mechanisms. This combination therapy strategy could enhance immune responsiveness and may help overcome limitations associated with immunotherapy in solid tumors.

Future studies will aim to determine whether combination treatment can suppress β-catenin nuclear translocation and restore chemosensitivity in FAT1-deficient breast cancer cells. Besides, although PM reflects the active form of CTX, it bypasses the metabolic activation process and *in vivo* studies may be needed to validate its clinical relevance.

## Material and Methods

### For studies with human subjects

This study was approved by the Ethics committee of Renji Hospital, School of Medicine, Shanghai Jiao Tong University and the ethics number is *KY2023-115-C*. All patients were given informed consent for sample collection.

### Database

#### cBioportal (http://www.cbioportal.org/)

We analyzed features of *FAT1* across various oncologic cohorts using the cBioportal database 56. The mutation frequency of *FAT1* was examined in the breast cancer cohort with 1,365 samples (MSK, Cancer Discovery 2022). Additionally, we analyzed *FAT1* mutations in a pan-cancer context—including oncoprints, cancer type summaries, mutation data, and survival data based on 32 selected studies comprising 10,967 samples from the TCGA PanCancer Atlas.

#### UALCAN (https://ualcan.path.uab.edu/)

The differential expression analysis of *FAT1* and survival prognosis in breast cancer was searched using the UALCAN database 57: 1) On top of the homepage, "TCGA analysis" option was selected; 2) The gene symbol *FAT1* was entered in the "Enter gene symbol(s)" field; 3) Tumor type “Breast invasive carcinoma” was selected to view the differential expression, survival rate and associated statistical data.

#### GEPIA2 (http://gepia2.cancer-pku.cn/#index)

Correlation between FAT1 and CTNNB1 expression in breast cancer was assessed using the GEPIA2 platform 58: 1) On the left of the page, select “Correlation Analysis”; 2) “Gene A” was set to *FAT1* and “Gene B” to *CTNNB1*; 3) The dataset “BRCA Tumor” was chosen, and “Pearson” was selected as the statistical method.

#### STRING (https://string-db.org/)

PPI involving FAT1 were explored using the STRING database investigated the interactions between different proteins 59: 1) Entering FAT1 in the “Protein Name” field and selecting "Homo sapiens" as the species; 2) Clicking on “Legend” to interpret the color-coded interaction network and corresponding score values.

### Methods

#### Breast cancer tissue dissociation

Surgically resected samples were immersed into tissue storage solution within tubes and kept on ice (0°C) during transport. Upon arrival at the laboratory, fresh tissues were transferred into a sterile dish on ice, where dissection tools were used to carefully remove fatty tissue, calcifications, blood clots, and necrotic tissue. Each sample was divided into four parts for transcriptomic sequencing, genomic sequencing, histological examination, and organoid establishment.

The tissues were washed twice in ice-cold washing buffer containing PBS (Hyclone, SH30256.01) and 2% P/S (Gibco, 15070063), until the supernatant was clear. Subsequently, the cleaned tissues were cut into small pieces (1-3 mm³) and incubated with digestion buffer (Advanced DMEM/F12 (Gibco, 12634010), 1 mg/mL Collagenase IV (Gibco, 17104019), 0.1 mg/mL DNaseI (Sigma Aldrich, 9003-98-9)). The volume of digestion buffer added was at least twice the volume of tissue. Pre-treated tissues were transferred to centrifuge tubes and incubated on a shaker at 37°C for 30-90 minutes, until most of bulk tissues had been dissociated into single-cell suspensions. Digestion was halted by adding stop buffer (Advanced DMEM/F12 supplemented with 2% BSA; Yeasen, 36101ES), followed by filtration through a 40-μm cell strainer and centrifuged at 200×g for 5 minutes. If the cell pellet appeared red, it was resuspended in 1 mL RBC lysis buffer (Beyotime, C3702) and incubated on ice for 2 minutes. The cells were then washed twice with washing buffer.

#### Organoids culture and passage

Pre-treated cell clusters (2-3×10^5^ cells) were resuspended in 10 μL ice-cold Matrigel (Corning, 356234) in a 1.5 mL tube at a 1:1 ratio. The cell-Matrigel mixture was then seeded as 10 μL droplets onto a pre-warmed 48-well plate (Corning) and incubated in a humidified 37°C incubator with 5% CO_2_ for 10-15 minutes to allow gelation. Subsequently, 250 μL of culture medium was added to each well. The medium was prepared according to previously established protocols 16, 60 and refreshed approximately every 3 days. Organoids were passaged based on density and size.

For passaging, organoids were harvested and resuspended in 300 μL TrypLE (Corning, 12605028) by gently pipetting and incubated at 37 °C for 4-8 minutes, with additional pipetting to dissociate cell clusters into single cells. After digestion, 500 μL of Advanced DMEM/F12 was added, and then centrifuged at 200×g for 5 minutes. The cell pellet was resuspended in ice-cold Matrigel at appreciate ratios (typically 1:1 to 1:6) for re-seeding.

#### Organoid formation assay, growth size and proliferation index analysis

For the organoid formation assay, 2×10^3^ cells were mixed with 2 μL ice-cold Matrigel at a 1:1 ratio and seeded as domes onto a pre-warmed 96-well plate (Corning). Organoids were allowed to develop from single cells over an 8-day period, with their growth sizes tracked every 2 days using an optical microscope (Motic, AE31E). Number of organoids formed (>50 µm) were counted using ImageJ (v.15.3), and their diameters were measured by Adobe Illustrator 2021.

Proliferation index was assessed by quantifying the percentage of Ki67-positive cells, identified by detecting by the brown coloration in immunostaining images 61. Images were deconvolved using the Colour Deconvolution for hematoxylin and DAB, converted to 8-bit binary images and analyzed using the IHC Toolbox to evaluate the ratio of Ki67-positive cells to total cells. All data were summarized and analyzed using Prism 9.

#### Histology and Immunohistochemistry

Organoids were carefully detached from the culture plate and embedded in low-melting-point agarose (Sigma Aldrich, A9045-25G). Both patient-derived tissues and embedded organoids were fixed in 4% paraformaldehyde (LABLEAD, P4500) followed standard protocols, including dehydration, paraffin embedding, sectioning and staining. Paraffin sections with a thickness of 10 μm were prepared for all analyses. Histological and immunohistochemical staining were performed using Fully Automated Research Stainer (Leica, BOND RX) and an Integrated Workstation (Leica, ST5010/TS5015/CV5030), in accordance with the manufacturer's instructions. The primary antibodies involved in this study included ER (Abcam, ab32063, 1:500), PR (Abcam, ab16661, 1:500), HER2 (Abcam, ab134182, 1:1000), Ki67 (Abcam, ab16667, 1:1000). Images were acquired using an Olympus BX43 microscope.

#### Drug screening test

The effect of drugs on organoids were assessed using the cell counting kit-8 (CCK-8) assay and measured by OD_450_ values. Organoids were dissociated into single cells as previously described. Approximately 2×10^3^ cells resuspended in 2 μL Matrigel and seeded into 96-well plate (Corning) with 70 μL of culture medium for 5 days before drug treatment. Immediately before use, the CCK-8 (Selleck, B34304) reagent was diluted in Advanced DMEM/F12 at a 1:9 ratio. After carefully discarding the culture medium, 100 μL freshly prepared CCK-8 solution was added to each well. Plates were incubated at 37 °C for 1 hour and OD₄₅₀ values were measured using a microplate reader (Bio-Tek, Synergy HTX). After 3 days of treatment with different drug concentrations, cell viability was reassessed using the same procedure. All data were normalized to negative control (DMSO), with positive control (Puromycin) and blank control (culture medium only) included for comparison. The drugs used in this study included: Doxorubicin (Shyuany, 25316-40-9), Olaparib (MCE, HY-10162), Palbociclib (MCE, HY-50767) and Phosphoramide mustard cyclohexanamine (active metabolite of CTX; MCE, HY-137316A).

For cell lines, 2×10^3^ cells per well were seeded into a 96-well plate (Corning) and cultured for 24 hours. Before drug treatment, 100 μL CCK-8 working solution (1/10 volume of culture medium) was added and baseline OD_450_ values were recorded. Samples were then washed twice with PBS (HyClone, SH30256.01) and fresh culture medium containing respective drug concentrations was added. After 48 hours of drug exposure, OD_450_ values were measured again and compared to baseline. Data were normalized to negative control (DMSO), with positive control (PBS only) and blank control (culture medium only).

#### Cell viability and imaging

Cell viability was assessed using the Calcein AM/PI Cytotoxicity Assay Kit (Beyotime, C2015S, 1000×), which provided dual fluorescence staining to distinguish live and dead cells. Fresh working buffer (1×) was prepared following the manufacturer's protocol. The supernatant from wells containing the organoids was carefully removed, followed by two washes with PBS. Organoids were stained with 100 μL of the working buffer (1×), incubated at room temperature for 30 minutes in the dark. After staining, organoids were gently washed twice with PBS and then 100 μL PBS was added prior to imaging. Fluorescence imaging was conducted using a fluorescence microscope (Nikon, ECLIPSE Ts2). Live cells were labeled with green fluorescence (Calcein AM), while dead cells exhibited red (PI). Fluorescence intensity was further quantified using ImageJ (v1.53a) and the plot profiles were exported using Microsoft Excel (v16.74).

### Whole exome sequencing (WES) analysis

#### DNA extraction

DNA was extracted from all samples using Genomic DNA Purification Kit (EZB, B0007), following manufacturer's protocols supplied with the kit. DNA purification and concentration were assessed by NanoDrop spectrophotometers (Thermo, 2000c).

#### Library construction and sequencing

Library preparation and sequencing were performed by Mingma Technologies Co., Ltd (Shanghai, China). A total of 200 ng genomic DNA was fragmented using the Agilent's SureSelect Enzymatic Fragmentation Kit for ILM, targeting an average fragment size of 150-200 bp. Libraries were prepared with the SureSelect XT HS2 Reagent Kit (Agilent), and adapter-ligated DNA fragments were amplified using Herculase II Fusion DNA Polymerase (Agilent). Pre-capture libraries containing exome sequences were captured with SureSelect HS Human All Exon V8 (Agilent). Library concentration was quantified using a Qubit 3.0 fluorometer dsDNA HS Assay (Thermo Fisher Scientific), and size distribution was analyzed using the Agilent Bioanalyzer 4200 (Agilent). Paired-end sequencing was performed using the NovaSeq 6000 S4 Reagent Kit v1.5 (300 cycles) on the Illumina NovaSeq 6000 platform (Illumina, San Diego, USA).

### Data analysis

Raw sequencing data underwent quality control using FastQC (v0.11.8). Adapter trimming was conducted with Trim Galore (v0.5.0). Clean reads were aligned to the human reference genome GRCh38 using Burrows-Wheeler Alignment with maximal exact matches (BWA-MEM) (v0.7.17). Alignment statistics were summarized using MultiQC (v1.7) 62. SAMtools (v0.1.9) was utilized for sorting alignment files and indexing BAM files 63. Data preprocessing followed the GATK Best Practices using Genome Analysis ToolKit (GATK, v4.1.1.0) 64, including base quality score recalibration and variant calling. Mutational signature analysis was performed with MutationalPatterns (v3.14.0) to calculate the optimal contribution of COSMIC signatures and determine the genomic context for all somatic SNVs in both tumor tissues and PDOs 15.

### Bulk RNA sequencing analysis

#### RNA extraction

Total RNA was extracted using the RNeasy Mini Kit (Qiagen, 74106) following manufacturer's instruction. RNA concentration and purity were assessed using a NanoDrop spectrophotometers (Thermo,2000c). Samples were stored at -80 °C for downstream applications, avoiding freeze-thaw cycles.

#### Library construction and sequencing

Library construction and sequencing were conducted by Mingma Technologies Co., Ltd (Shanghai, China). To construct sequencing libraries, 500 ng of high-quality RNA (OD260/280=1.9~2.0, RIN≥8) was required. mRNA-focused sequencing libraries were prepared from total RNA using the VAHTS mRNA-seq v3 Library Prep Kit (VAHTS, NR611). PolyA mRNA was isolated with oligo-dT-attached magnetic beads and fragmented. First-strand cDNA was synthesized using reverse transcriptase and random primers, followed by second-strand synthesis. The cDNA was then end-repaired, phosphorylated, and had 'A' bases added as per Illumina's protocol. Illumina sequencing adapters were ligated to both ends of the cDNA fragments. After PCR amplification, target fragments (200-300 bp) were purified using CleanNGS (CleanNA-CNGS-0500). Post-library construction was quantified using a Qubit 3.0 fluorometer dsDNA HS Assay (Thermo Fisher Scientific), and size distribution was analyzed using an Agilent BioAnalyzer (Agilent). Sequencing was performed on an Illumina system following the manufacturer's protocols.

#### Data analysis

RNA-sequencing data analyses and visualization were performed in R (v4.2.3). Differentially expressed genes (DEGs) between drug-sensitive and drug-resistant PDOs were identified by the edgeR package (v3.26.8) 65. DEGs were filtered using thresholds of FDR ≤ 0.01 and FC ≥ 2), and mapped to cancer-related signaling pathways. Gene set enrichment analysis (GSEA) and gene ontology (GO) enrichment were performed by GSEA (v1.2) with gene sets obtained from MSigDB (v7.5.1) 66-68. Gene set variation analysis (GSVA) (v1.46.0) was used to assess pathway activation differences between the resistant and the sensitive group 69.

### Cell culture, passage and transfection

Human breast cancer cells, MCF-7 (RRID: CVCL_0031; female) and MDA-MB-231 (RRID: CVCL_0062; female) were kindly provided from Dr. Aina He, Sixth People's Hospital Affiliated to Shanghai Jiaotong University School of Medicine. Short tandem repeat (STR) genotyping of cell lines was completed by Shanghai Biowing Biology (Shanghai, China, Report number: NO.20220707-STR05).

MCF-7, MCF-7 derived cells (MCF-7 FAT1#1/2/3 and blank), MDA-MB-231 and MDA-MB-231 derived cells (MDA-MB-231 FAT1#1/2/3 and blank) were maintained in RPMI 1640 (Yeasen, 41402ES76), supplemented with 10% FBS (Procell, 164210), 1% P/S (Gibco, 15070063). Cells were cultured in incubator (Thermo) at 37°C with 5% CO_2_.

Cell Passaging was performed using standard method. Briefly, cells at high confluence (80%-90%) were digested with 0.25% Trypsin-EDTA (Yeasen, 40127ES60) for 3 minutes at 37°C, and then stopped by adding culture medium. The cell suspension was transferred into 15 mL tubes (Corning) and centrifuged at 400×g at room temperature for 5 minutes. Cells were then passaged at a 1:3 to 1:4 ratio in 10 cm culture dishes (Corning).

For stable knockdown of FAT1, lentiviruses were ordered from GeneChem (Shanghai, China), constructed in GV493 (hU6-MCS-CBh-GFP-IRES-Puromycin) vectors. Before transfection, cells were seeded in 6-well plates (Corning) for 24 hours under standard 2D culture conditions until reaching 20%-30% confluence. The next day, 1 mL of virus infection solution containing virus (sh1/2/3 and blank) and 40 µL HitransG P (25×, GeneChem) was prepared in anti-free culture medium respectively. The virus volume was calculated using the formula: Virus volume = (MOI × Number of cells) / Virus titer (1×10^8^ TU/mL). The MOI was set to 20 for MCF-7 cells and 10 for MDA-MB-231 cells. Control wells received medium containing only 40 μL HitransG P. After 16 hours of incubation, the viral supernatant was removed and replaced with 1 mL of fresh culture medium. After 72 hours, green fluorescent protein (GFP) was observed under a microscope (Nikon, ECLIPSE Ts2) to confirm successful transfection. Cells exhibiting healthy morphology and approximately 80% GFP positivity were selected for puromycin selection using 2 μg/mL puromycin (Sangon, E607054), until control cells were almost eliminated. Successfully established cell lines were maintained for subsequent experiments.

### Cell proliferation assay

Cell viability was assessed using the CCK-8 assay (Selleck, B34304) as previously described. Briefly, all cell lines (8×10^2^ / per well) were seeded in 96-well plates (Corning) and cultured in full medium. After 24 hours, 100 μL of fresh CCK-8 working solution (1/10 volume of culture medium) was added and the plates were incubated at 37°C for 1 hour. After acquiring OD_450_ values, the cells were washed with PBS (HyClone, SH30256.01) containing 2% P/S (Gibco, 15070063) and fresh culture medium was added for further culture. Cell viability was tracked every 2 days on days 1, 3, 5 and 7.

### Western blotting

Cells were lysed by RIPA (Beyotime, P0013C) supplemented with protease (Bimake, B14001) and phosphatase (Bimake, B14001) inhibitor cocktail. Proteins concentrations were quantified with a BCA protein assay kit (Beyotime, P0012S) and samples were diluted in loading buffer (Beyotime, P0286) to a final concentration of 2 µg/µL.

Equal amounts of protein were loaded onto 6% SDS-PAGE gels (Epizeme, PG110) for FAT1 and 10% gels (Epizeme, PG112) for CTNNB1. After performing electrophoresis, separated proteins were transferred to 0.22 µm PVDF membranes (Merck Millipore). The membranes were blocked with protein free rapid blocking buffer (Epizyme, PS108P) for 10 minutes at room temperature, followed by overnight incubation at 4 °C with the following primary antibodies: GAPDH (Abcam, ab291253, 1:10000), FAT1 (Abcam, ab190242, 1:2000) and CTNNB1 (Abcam, ab32572, 1:2000). Membranes were washed three times with PBST (PBS containing 0.1% Tween-20), then incubated for 1 hour at room temperature on a shaker with the corresponding second antibodies: Anti-rabbit, (Beyotime, A0208, 1:2000); Anti-mouse, (Beyotime, A0216, 1:2000). All antibodies were diluted in universal antibody diluent (Epizyme, PS119). Finally, the membranes were exposed by enhanced chemiluminescence solution (Thermo Fisher Scientific) and images were captured with a Gel Doc EZ Imager (BIO-RAD). Analysis of images were performed using Image Lab software (BIO-RAD).

### Co-immunoprecipitation (Co-IP)

Co-IP was performed to assess the interaction between FAT1 and CTNNB1. Collected cells were lysed by cell lysis buffer for Western and IP (Beyotime, P0013). The 50 μL of Protein A/G magnetic beads (Selleck, B23201) were incubated with CTNNB1 antibody (Abcam, ab32572,1:30) to immunoprecipitate the target complex. The antibody-conjugated beads were then incubated with cell lysates overnight at 4 °C. Immunoprecipitates were eluted by boiling in SDS loading buffer (Beyotime, P0286) and separated by SDS-PAGE followed by Western blotting.

### Immunofluorescence

For organoids, the slides were prepared as described in the *Histology and Immunohistochemistry* methods section. Sections were blocked with 5% BSA (Yeasen, 36101ES) for 1 hour at room temperature. Primary antibodies (beta catenin, abmart, T53523S; E-cadherin, abmart, TA0131S) were applied and incubated overnight at 4°C. The next day, sections were washed with PBS and incubated with secondary antibodies for 1 hour at room temperature in the dark. Nuclei were counterstained with DAPI (1:1000, Invitrogen, D1306), and fluorescence images were acquired using a Leica laser scanning confocal microscope.

For cell lines, cells were seeded on coverslips in a 12-well plate (Corning) at a density of 2×10^3^ cells per well. Next day, cells were fixed with 4% paraformaldehyde (LABLEAD, P4500) for 15 minutes and permeabilized with 0.5% Triton X-100 (Sigma Aldrich, 9036-19-5) for 20 minutes. The fixed cells were then blocked with 5% BSA (Yeasen, 36101ES) for 1 hour at room temperature. Primary antibodies (1:250, abcam, ab32572) were added and incubated with the cells overnight at 4°C. Subsequently, secondary antibodies were incubated at room temperature for 1 hour in the dark. Finally, the nucleus was stained with DAPI (1:1000, Invitrogen, D1306) and images were captured using a laser scanning confocal microscope (Leica). Image visualization was performed using SlideViewer software (CaseViewer 2.5, 64-bit version).

### Quantitative real-time reverse transcription PCR (qRT-PCR)

Total RNA was extracted using the RNeasy Mini Kit (Qiagen, 74106). Reverse transcription was performed using the cDNA Synthesis Kit (EZBioscience, A0012-R2). Subsequently, qRT-PCR was performed using SYBR Green qPCR Master Mix (A0012-R2, EZB) on the QuantStudio™ 7 Flex Real-Time PCR System (ABI-Q7, Thermo). Relative gene expression levels were calculated using the 2^-ΔΔCt^ method was used to analyze the relative changes, with GAPDH as the control. Primer sequences used in this study are listed in Table 1.

### Statistical analysis

Statistical analyses were performed using GraphPad Prism 9. To evaluate drug sensitivity, cell viability data from dose-response experiments were used to calculate half-maximal inhibitory concentration (IC₅₀) values. The OD₄₅₀ values were normalized to the negative control (DMSO), and the data were fitted using a nonlinear regression model.

For comparisons between two groups, a two-tailed t-test was applied. For multiple group comparisons, one-way ANOVA followed by Tukey's post hoc test was used. Differences were statistically significant at *P* < 0.05 and significance values are indicated as * *P* < 0.05, ** *P* < 0.01, *** *P* < 0.001, **** *P* < 0.0001.

## Supplementary Material

Supplementary figure.

## Figures and Tables

**Table 1:** Primer sequences of qRT-PCR used in this study.

Gene	Forward (5'-3')	Reverse (5'-3')
FAT1	CATCCTGTCAAGATGGGTGTT	TCCGAGAATGTACTCTTCAGCTT
CTNNB1	AAAGCGGCTGTTAGTCACTGG	CGAGTCATTGCATACTGTCCAT
AES	ACCCCAGCAACTCAAATTCAC	AAGCCGTAGGACATCTCGTAG
AXIN1	GGTTTCCCCTTGGACCTCG	CCGTCGAAGTCTCACCTTTAATG
DVL1	GAGGGTGCTCACTCGGATG	GTGCCTGTCTCGTTGTCCA
GSK3A	GGAAAGGCATCTGTCGGGG	GAGTGGCTACGACTGTGGTC
LLGL1	CTGTCACACAGATGCACTTCT	GCCATTATGGTGGACAATCTCC
TCF3	ACGAGCGTATGGGCTACCA	GTTATTGCTTGAGTGATCCGGG
TCF7L1	TCGTCCCTGGTCAACGAGT	ACTTCGGCGAAATAGTCCCG
GAPDH	GGAGCGAGATCCCTCCAAAAT	GGCTGTTGTCATACTTCTCATGG

## Abbreviations

BC: Breast cancer
CTX: Cyclophosphamide
CKI: CDK 4/6 inhibitor
PARPi: PARP inhibitor
FAT1: FAT Atypical Cadherin 1
PDOs: Patient-derived organoids
H&E: Haematoxylin and eosin
IHC: Immunohistochemical
ER: Estrogen receptor
PR: Progesterone receptor
HER2: Human epidermal growth factor receptor 2
WES: Whole exome sequencing
SNPs: Single-nucleotide polymorphisms
CNVs: Copy number variations
PM: Phosphoramide mustard
IC₅₀: Half maximal inhibitory concentration
GO: Gene oncology
GSEA: Gene set enrichment analysis
FAT1-WT: FAT1 wild-type
FAT1-Mut: FAT1 mutant
FAT1-KD: FAT1-Knowdown
OS: Overall survival
PFS: Progression-free survival
DSS: Disease-specific survival
PPI: Protein-protein interaction
GSVA: Gene set variation analysis
DST: Drug screening tests
qRT-PCR: Quantitative real-time reverse transcription PCR
