# Supplementary material for: Loss of FAT1 drives cyclophosphamide resistance in breast cancer via the Wnt/β-Catenin pathway
Source: Int J Biol Sci. 2026 Jan 1;22(1):447–65. doi: 10.7150/ijbs.117161 (PMC12681942; doi:10.7150/ijbs.117161)
Supplement: Supplementary file 1 — Supplementary figure. [file ijbsv22p0447s1.pdf]

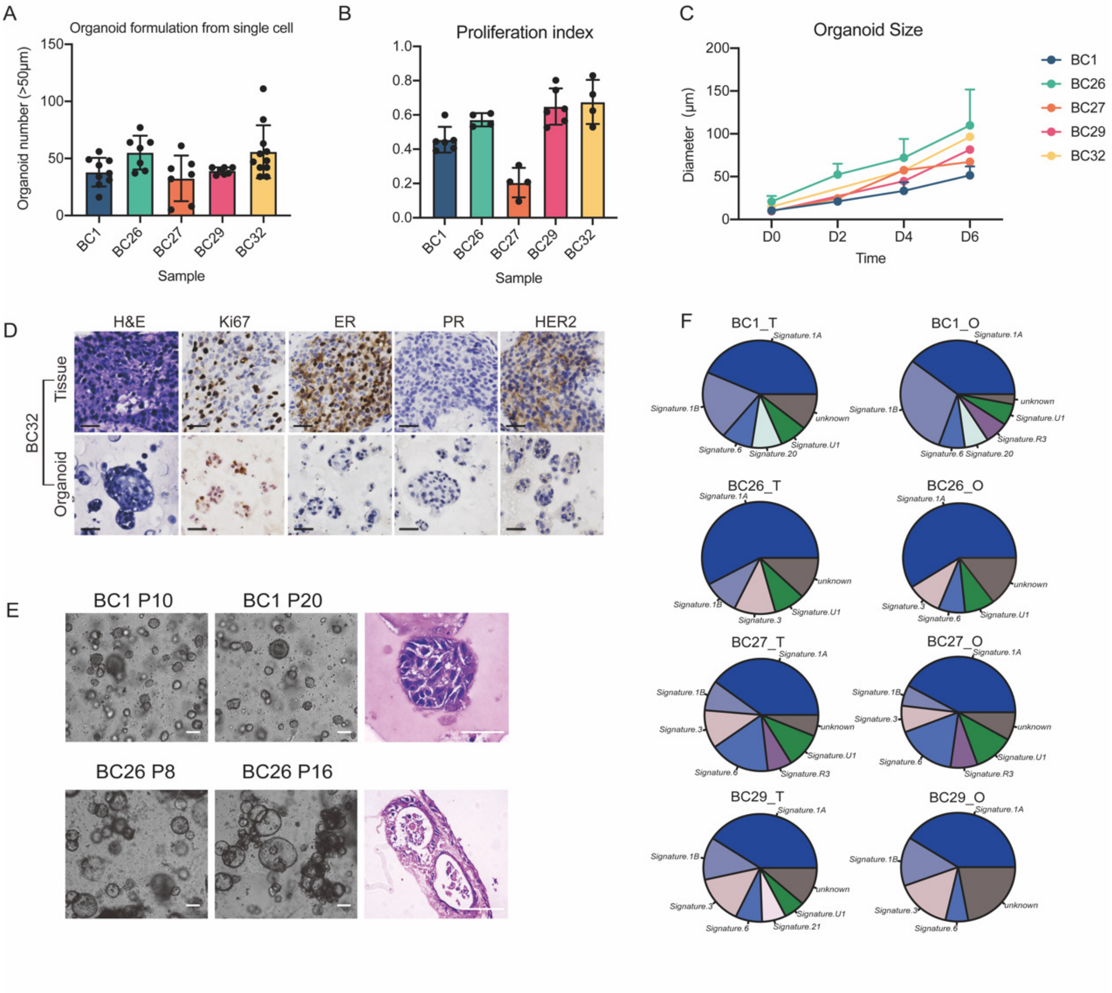

Fig. S1. PDOs maintain tumor heterogeneity. (A) Number of organoid formulations. Error bars: ±s.d, n≥6. (B) Proliferation index of each BC-PDO. Error bars: ±s.d, n ≥4. (C) Diameter size of PDOs from day0 (D0) to day6 (D6). Error bars: ±s.d, n=4. (D) H&E and IHC staining displayed molecular features. Scale bar: 100 μm. (E) PDOs maintained histological features after long-term passages. Scale bar: 100 μm. (F) PDOs maintained genomic signatures after long-term passages.
